# Management Outcomes in Pediatric Keratoconus: Childhood Keratoconus Study

**DOI:** 10.1155/2022/4021288

**Published:** 2022-02-07

**Authors:** Yogita Gupta, Rohit Saxena, Vishal Jhanji, Prafulla K. Maharana, Rajesh Sinha, Tushar Agarwal, Jeewan S. Titiyal, Namrata Sharma

**Affiliations:** ^1^Dr. Rajendra Prasad Centre for Ophthalmic Sciences, All India Institute of Medical Sciences, Delhi, India; ^2^University of Pittsburgh School of Medicine, Pittsburgh, Pennsylvania, USA

## Abstract

**Introduction:**

The Global Delphi Panel of Keratoconus (KC) and Ectatic Diseases formulated management guidelines for KC in 2015. The aim of this study was to evaluate management outcomes in pediatric KC.

**Materials and Methods:**

Prospective, interventional study was conducted at a tertiary care hospital including KC patients aged <18 years. Based on disease severity and progression of disease, patients were prescribed either glasses or contact lenses (CLs) or underwent corneal collagen crosslinking (CXL), deep anterior lamellar keratoplasty (DALK), or penetrating keratoplasty (PK). *Main Outcome Measures*. Best corrected visual acuity (BCVA), manifest cylinder, maximum keratometry, thinnest corneal thickness, total higher order aberrations, and corneal hysteresis at baseline and 12 and 24 months.

**Results:**

116 eyes of 62 patients with a mean age of 14.76 ± 2.77 years were included. 32.8% of the eyes (*n* = 38) achieved satisfactory BCVA with glasses/CLs only. Corneal collagen crosslinking (CXL) was performed in 43.1% of the eyes (*n* = 50) with progressive KC and halting of progression was noted in 83.3% (*n* = 35) of the eyes at 2 years. 7.7% of the eyes (*n* = 9) were managed for acute hydrops. DALK and PK were successfully performed in 9.5% (*n* = 11) and 6.9% (*n* = 8) of the eyes with BCVA of 0.14 ± 0.09 and 0.08 ± 0.12 at 2 years, respectively.

**Conclusions:**

Pediatric KC cases with progression show good visual and aberrometric outcomes and halting of progression after CXL. DALK and PK have good outcomes. The global consensus guidelines showed good clinical utility in pediatric patients. Presence of VKC did not have an impact on the outcomes of CXL in pediatric patients.

## 1. Introduction

Keratoconus (KC) is an ectatic condition of the cornea, associated with progressive corneal thinning. It induces corneal astigmatism and leads to visual impairment. Keratoconus, classically, has its onset at puberty and is usually progressive until the third to fourth decade of life [1, 2]. Last two decades have seen an emergence of new knowledge leading to a better diagnosis and management of KC. Worldwide, its prevalence is about 1.38 per 1000 [3] and incidence is nearly 50–230 cases per 100000 population [4] and it is reportedly much high in the Middle-Eastern and Asian countries due to ethnic differences [5, 6].

In 2015, Gomes et al. reached a global consensus regarding diagnosis and management of KC using a Delphi method involving 36 expert panelists [7, 8]. Agreements reached upon nonsurgical management of KC were a verbal guidance to patients to avoid rubbing, use of topical antiallergic medications, use of preservative-free lubricants, and use of glasses or scleral, hybrid, and gas-permeable contact lenses (CLs) for visual rehabilitation [7]. The panel consensus for surgical management suggested corneal collagen crosslinking (CXL) in patients with perceived risk of progression (i.e., clinical progression has not been confirmed) and in young patients (∼15 years of age) with progressive KC even with satisfactory vision with glasses, followed by glasses/CLs [7]. For KC eyes without the evidence of progression, there was no consensus on whether there is an age below which CXL may be performed or on whether any uncorrected visual acuity better than which CXL may be restricted [7]. For cases with stable KC but unsatisfactory vision with glasses/CLs or intolerant to CLs, deep anterior lamellar keratoplasty (DALK) or penetrating keratoplasty (PK) was recommended [7]. Intracorneal ring segments (ICRS) were suggested as a treatment option in stable KC with adequate corneal thickness and minimal scarring but unsatisfactory vision with glasses/CLs [7].

Nearly half of the cases of pediatric KC are more advanced at the time of presentation compared to adult KC [9]. It also progresses at a faster rate [9, 10] and therefore early detection and close monitoring are advocated in pediatric KC [10]. Pediatric KC is also associated with vernal keratoconjunctivitis (VKC) [9]. Eye rubbing and chronic inflammation in cases of VKC can lead to increased severity of KC [9, 11] and an increased risk of complications such as corneal hydrops [12]. While studies evaluating adult KC have reported good outcomes using these Global Consensus guidelines, there is inadequate information regarding pediatric KC. The aim of this study was to evaluate management outcomes in pediatric keratoconus (KC).

## 2. Methods

This prospective interventional nonrandomized clinical trial called “Childhood Keratoconus Study” is a study registered with the Clinical Trials Registry Institute, India (CTRI/2017/05/008578, India), and approved by the ethics committee of the institute. The study protocol adhered to the tenets of the Declaration of Helsinki. A written, informed consent was obtained from the parents/legally authorized representatives (LARs) of all participants for participation, intervention, and follow-up. In addition, an assent form was filled by all adolescent participants above 12 years of age. The described protocol for diagnosis and management of KC was based on the recommendations of the Global Consensus on Keratoconus and Ectatic disorders [7, 8].

### 2.1. Participants

All cases of unilateral/bilateral KC with age <18 years were included in the study. Definition of childhood (age <18 years of age) was adopted from the United Nations High Commissioner of Human Rights. The following patients were excluded from the study: patients with parents/LARs not willing to give consent for participation/follow-up and any surgical intervention done in the eye previously or associated systemic conditions such as asthma, eczema, or allergic rhinitis.

Diagnosis of KC was based on the definition given by Global Consensus on Keratoconus and Ectatic disorders (abnormal posterior elevation, abnormal corneal thickness distribution, and clinical noninflammatory corneal thinning). Diagnosis of VKC was based on the presence of severe itching with a history of seasonal exacerbation, ropy discharge, and slit lamp signs such as conjunctival hyperemia, papillary hypertrophy (0.1 to 5 mm diameter) in tarsal conjunctiva, giant papillae, Horner-Tranta's dots on the limbus, and/or limbal papillae or limbal infiltrates. Severity of VKC was graded using criteria described by Bonini et al. [13] as quiescent, mild, moderate, severe, very severe, and evolution grades. KC progression was defined as in global consensus guidelines as a consistent change in at least two of the following parameters where the magnitude of change is above the normal noise of the testing system: progressive steepening of the anterior corneal surface (elevation on anterior float map), progressive steepening of the posterior corneal surface (elevation on posterior float) and progressive thinning (thinnest pachymetry), and/or an increase in the rate of corneal thickness change from periphery to the thinnest point (pachmetry progression indices), verified by two consecutive examinations at 3-month interval.

### 2.2. Patient Evaluation

All patients underwent a detailed clinical evaluation to determine disease severity. Cases were grouped into VKC (VKC group) and non-VKC (non- VKC group) as well as into progressive and nonprogressive KC (verified in a 3-month observation period). KC severity was assessed using the Amsler– Krumeich classification system. Uncorrected visual acuity (UCVA) and best corrected visual acuity (BCVA) were measured on Snellen's vision charts. Manifest refraction was performed to measure spherical equivalent (SE) and astigmatism (ASTIG). Values of maximum (Kmax), steep (Ksteep) and flat keratometry (Kflat), central corneal thickness (CCT), thinnest corneal thickness (thCT), and endothelial cell density (ECD) were noted at baseline. *Cone location* (central and paracentral cones defined as ectasia on posterior surface on the enhanced ectasia display within <3 mm zone and 3-5 mm zone, respectively, from center of the pupil) was noted from the Pentacam (Oculus Inc., Wetzlar, Germany). Root mean square (RMS) values of corneal higher order aberrations (HOA) were noted from corneal aberrometer (iTRACE Surgical Workstation Hoya Surgical Optics, Chino Hills, Calif.). The magnitudes of corneal hysteresis (CH) and corneal resistance factor (CRF) were obtained from Ocular Response Analyzer (ORA, Reichert Ophthalmic instruments, Buffalo, NY, USA). Confocal microscopy was performed to measure stromal keratocyte density in anterior, middle, and posterior corneal stroma (ConfoScan 4, Nidek Technologies, Inc., Greensboro, North  Carolina, USA).

### 2.3. Intervention

A severity-based management approach was followed for all cases according to the global consensus guidelines. Cases of acute hydrops were managed with intracameral gas injection, with supporting medications: topical sodium chloride (5%) QID and topical carboxy-methylcellulose sodium drops (1%, Refresh Liquigel, Allergan Inc., Irvine, CA) QID for three weeks. If vision was not satisfactory, surgical options like deep anterior lamellar keratoplasty (DALK), penetrating keratoplasty (PK), or intrastromal corneal ring segment (ICRS) were considered. Accelerated CXL (ACXL) was performed for progressive keratoconus using Avedro KXL™ System (Avedro, Waltham, Mass., USA), followed by rehabilitation with glasses or contact lenses (CLs). Patients with associated VKC underwent CXL only after adequate control of inflammation (defined as no signs or symptoms for a period of at least three months prior to CXL), with the use of topical steroids (0.1% fluorometholone eye drops) and topical antiallergics (0.1% olopatadine hydrochloride), cyclosporine eye drops (0.05%), and/or tacrolimus eye ointment (0.03%). Adequate inflammation control was defined as absence of symptoms, absence of conjunctival hyperemia, and noninflamed papillae for a minimum period of 3 months [13].

CXL was performed in the operating room under general anaesthesia. Epithelial debridement was performed in central 8 mm zone, followed by instillation of isotonic riboflavin (0.1% in 20% Dextran T500 medium) for 15 minutes with one drop every two minutes. UV-A light was used to irradiate the corneal surface for 5 minutes (365 nm; 18 mW/cm^2^) with continued riboflavin instillation. A bandage contact lens was placed at the end of the procedure. The postoperative treatment consisted of topical moxifloxacin hydrochloride drops (0.5%, Vigamox, Alcon Inc., Canada) three times a day for one week and topical carboxy-methylcellulose sodium drops (1%, Refresh Liquigel, Allergan Inc., Irvine, CA) QID for one month. Topical fluorometholone eye drops (0.1%, FML, Allergan Inc., Irvine, CA) were started after complete epithelialization: QID for one month and tapered thereafter over six weeks. Oral analgesics were given, if required.

### 2.4. Data Analysis

All data was managed in a Microsoft Excel spreadsheet. BCVA and UCVA were converted to logMAR. Statistical analysis was performed using R 3.4.4 (R Foundation, Vienna, Austria). Mean values for BCVA, CCT, thCT, ECD, SE, ASTIG, K flat, K steep, and K max were evaluated for VKC and non-VKC subjects by one-way repeated measures analysis of variance (ANOVA) which take into account correlation between fellow eyes. The mean values were compared between the two groups by Wald tests under two-way repeated measures ANOVA. Mean changes in the parameters were evaluated by one-way repeated measures ANOVA for each group and compared by Wald tests under two-way repeated measures ANOVA between the two groups. A *p*-value <0.05 was considered to be statistically significant. All categorical data was analyzed using Pearson Χ^2^ or Fisher's exact test. Spearman's rho correlation test (nonparametric) was used to study correlation of variables.

## 3. Results

**Figure 1:**
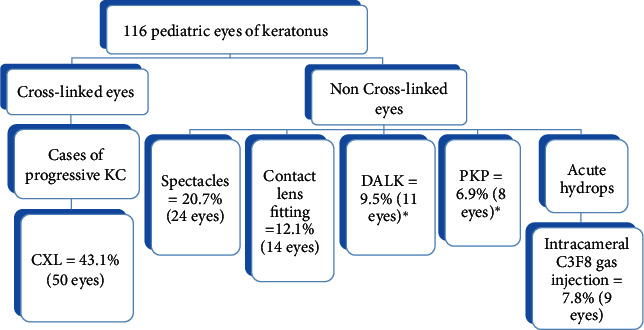
Management flowchart of cases of KC (^∗^DALK was planned in 12 eyes, but performed in 11 eyes as 1 eye had conversion to PKP due to intraoperative perforation of Descement's membrane; KC: keratoconus, CXL: corneal collagen cross linking, DALK: deep anterior lamellar keratoplasty, PKP: penetrating keratoplasty, and C3F8: octafluoropropane).

Figure 1 explains the management flowchart of the cases. A total of 116 eyes of 62 patients with a mean age of 14.76 ± 2.77 years (range, 8–18) were included (Table 1) [9]. The mean follow-up was 24.2 ± 1.3 months. 57 (92%) patients had bilateral presentation. Using Amsler–Krumeich staging, 58 eyes (50%) were classified as stage III or IV keratoconus. 62.9% of eyes had paracentral cones. 9 eyes (7.7%) presented with acute hydrops at presentation and required intracameral C3F8 injection. A total of 43.1% (*n* = 50) eyes underwent CXL. 9.5% (*n* = 11) eyes underwent DALK and 6.9% (*n* = 8) eyes underwent PK. Overall, 68 patients (58.6%) had some signs of VKC. There was no significant difference between age of patients in VKC and non-VKC groups (14.41 ± 2.79 vs. 15.1 ± 2.76 years, *p*=0.19). VKC group had more patients with grade 4 keratoconus (46% vs. 25%, *p*=0.004) with a higher mean *K* value (54.66 ± 7.97 vs. 50.68 ± 7.65 diopters, *p*=0.01), compared to non-VKC group.

### 3.1. Spectacles/CLs

CLs and glasses were dispensed to 12.1% (*n* = 14) and 20.7% (*n* = 24) of the patients, respectively, without any additional intervention. No significant changes were noted in BSCVA, UCVA, MRSE, and astigmatism at 12- and 24-month follow-up (Table 1). Rose-K2 lenses of Fluorolens 90 Blue lens design (Menicon Limited, Northampton, UK) with standard peripheral edge lift was the most commonly used contact lens. The contact lenses had a mean base curve of 6.86 ± 0.407 mm, mean diameter of 9.65 ± 0.95 mm, and mean power of -5.51 ± 3.14 D. After CL use, there was significant improvement in visual acuity from baseline mean BCVA of 0.23 ± 0.22 logMAR to 0.17 ± 0.6 logMAR at 24-month follow-up (*p*=0.001). A larger proportion of patients were intolerant to contact lens in the VKC group compared to the non-VKC group (41.1% vs. 29.1%, *p*=0.48).

### 3.2. DALK and PK

**Figure 2:**
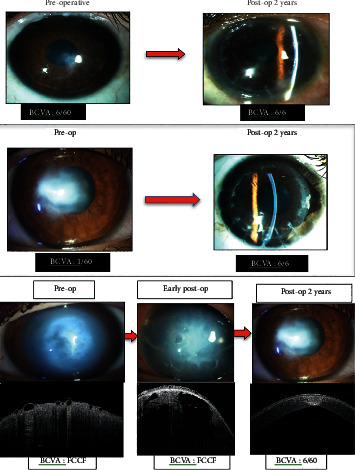
Preoperative and postoperative (2 years) photograph of a representative KC case that underwent (a) DALK, (b) PK, and (c) intracameral C_3_F_8_ gas injection in acute hydrops cases, with corresponding changes in AS-OCT image.

DALK was performed in 11 eyes (Figure 2(a)). At 24-month follow-up, there was a significant improvement in logMAR UCVA (from 1.53 ± 0.27 to 0.31 ± 0.04, *p* < 0.001), logMAR BCVA (from 1.24 ± 0.46 to 0.14 ± 0.09, *p* < 0.001), MRSE (from −6.1 ± 5.82, to −1.08 ± 1.16, *p*=0.001), and mean keratometry (from 64.31 ± 8.53 to 46.28 ± 7.55, *p* < 0.001). Eight eyes underwent PK (Figure 2(b)). There was a significant improvement in logMAR BCVA (from 1.54 ± 0.4 to 0.088 ± 0.12; *p* < 0.001), mean keratometry (from 66.3 ± 6.9 to 47.04 ± 0.95, *p* < 0.001) and spherical equivalent (from -8.45 ± 3.71 to -2.93 ± 1.25 D, *p*=0.004). The complications encountered in the DALK/PK group were intraoperative Descemet's membrane perforation (*n* = 2, 10.5%), graft epithelial problems (*n* = 1, 5.3%), subepithelial graft rejection (*n* = 1, 5.3%), high intraocular pressure (*n* = 1, 5.3%), and suture-related complications, including premature loosening (*n* = 1, 5.3%), broken sutures (*n* = 2, 10.5%), and suture-tract vascularization (*n* = 1, 5.3%).

### 3.3. Intracameral Gas Injection

Nine eyes presented with acute corneal hydrops and were managed with intracameral injection of perfluoropropane (C_3_F_8_) gas (Figure 2(c)) along with supporting topical medications. At 24 months, logMAR BCVA improved significantly from 1.84 ± 0.27 to 1.346 ± 0.52 (*p* < 0.001). After healing of hydrops, these patients were subsequently registered for corneal transplantation (DALK/PKP). The outcomes of these surgeries in healed hydrops cases were not reported in the current study.

### 3.4. Accelerated CXL

**Figure 3:**
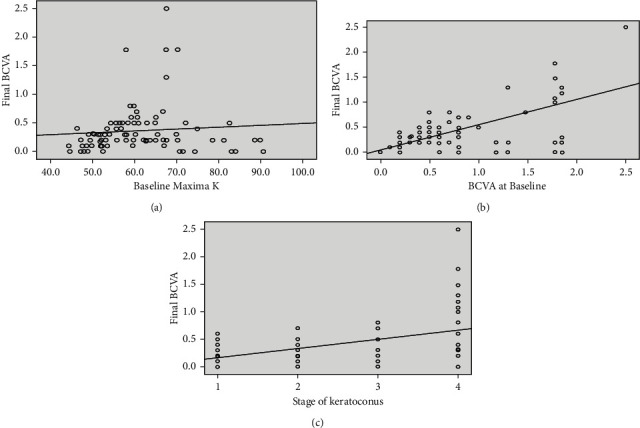
Significant positive correlation of BCVA at the final follow-up after CXL with (a) baseline BCVA (*p*=0.001), (b) baseline Kmax (*p*=0.001), and (c) stage of KC (*p*=0.01).

Accelerated CXL was performed in 43.1% of the eyes (*n* = 50). Mean age of the patients in this group was 14.26 ± 2.89 years (range: 8–18 years, *n* = 50). The BCVA improved significantly from 0.46 ± 0.24 logMAR at baseline to 0.38 ± 0.22 logMAR at 24 months' follow-up (*p* < 0.001, *n* = 50) (Table 1). A significant positive correlation was noted between final BCVA and baseline visual acuity (*r* = 0.809, *p* < 0.001), baseline keratometry (*r* = 0.66, *p* < 0.001), and severity of KC (*r* = 0.3,*p*=0.01) (Figure 3).

CXL was able to halt progression of KC in 35 eyes (83.33%) at the end of 2 years. Success of CXL at 2 years was noted to have no significant correlation with age (*r* = 0.215, *p*=0.17), presence of VKC (*r* = 0.108, *p*=0.494), stage of KC (*r* = 0.031, *p*=0.846), and HO total (*r* = 0.008, *p*=0.96) and a negative correlation with thinnest pachymetry at baseline (*r* = −0.016, *p*=0.921). Of the remaining 15 eyes, 6 eyes were managed with DALK/PK for progressive KC, 1 eye developed acute hydrops on 24-month follow-up, and 8 eyes developed complications after CXL: sterile infiltrates (4 eyes), infectious keratitis (1 eye), and mild corneal haze (3 eyes).

All keratometry values improved significantly at 24-month follow-up from baseline (*p* < 0.001 for all). The mean value of Kmean improved from 50.46 ± 4.31 D to 48.83 ± 4.62 D (*p* < 0.001, *n* = 42). Thinnest pachymetry reduced from 432.3 ± 45.64 *μ*m at baseline to 421.7 ± 48.65 *μ*m at 24-month follow-up after CXL (*n* = 42, *p* = 0.178). The mean central corneal thickness reduced from 438.5 ± 37.4 *μ*m at baseline to 436.7 ± 36.23 *μ*m at 24 months (*n* = 42, *p* = 0.011).

**Figure 4:**
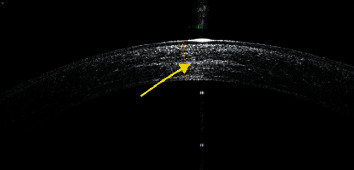
Anterior segment OCT image of the cornea showing demarcation line in post-CXL eye at 24-month follow-up at depth of 265 *μ*m.

Confocal microscopy was performed in 42 eyes. No significant changes were seen in endothelial cell density and keratocyte density in anterior, middle, and posterior corneal stroma at 24-month visit post CXL (Table 1). On ASOCT imaging, the mean depth of demarcation line (Figure 4) developing in the corneal stroma after CXL was noted at a depth of 292.02 ± 16.32 *μ*m and 287.11 ± 15.61 *μ*m at 12 and 24 months, respectively.

Corneal aberrometry was performed in 37 patients. The root mean square (RMS) values of total higher order aberrations (HO total) reduced significantly from 1.702 ± 1.56 *μ*m at baseline to 1.10 ± 1.19 *μ*m at 24 months after CXL (*p* < 0.001). RMS values of preoperative and postoperative trefoil changed significantly after CXL (from 0.82 ± 0.75 *μ*m at baseline to 0.45 ± 0.6 *μ*m at 24 months, *p* < 0.001), though coma showed no significant difference (from 1.007 ± 0.88 *μ*m at baseline to 0.95 ± 0.72 *μ*m at 24 months, *p*=0.11). There was no significant change in the value of CH (from 7.73 ± 1.44 mm Hg at baseline to 7.45 ± 1.41 mm Hg at 24 months, *p*=0.228, *n* = 42) and CRF (from 6.78 ± 1.5 mm Hg at baseline to 6.5 ± 1.38 mm Hg at 24 months, *p*=0.194, *n* = 42) after CXL.

Of all the cases undergoing CXL, 32 eyes (64%) had associated VKC and 18 eyes (36%) had no VKC. All 32 eyes had mixed (limbal and upper tarsal involvement) form of VKC and had quiescent stage [13] of VKC. Both groups showed significant improvement in Kmax, Kmean, UCVA, and BCVA after 2 years from baseline. However, there were no significant differences in the changes in these parameters in VKC vs. non-VKC groups.

### 3.5. Complications

The following complications were noted after CXL: sterile infiltrates (4 eyes), infectious keratitis (1 eye), and mild corneal haze (3 eyes). These were managed medically. No complications were noted in any other group.

## 4. Discussion

Management of any ocular disease in pediatric age group is challenging. Pediatric KC is one such challenging ocular condition. The average age at presentation of KC is around puberty. However, in Asian populations, it is known to have an earlier onset and a faster progression [14]. An increased prevalence of allergic eye diseases like VKC is also reported in these regions [15]. As such, KC is reported to be more severe in VKC eyes with reported thinner pachymetry [11, 12, 16], worse BCVA [11], and a higher rate of progression [17]. CXL may have complications in VKC eyes like flare up of inflammation and sterile or infectious keratitis, especially because it involves epithelial debridement.

CXL has proven efficacy in stopping the progression of KC over short term and long term [18–24]. In a long term prospective cohort study, Caporossi et al. demonstrated good efficacy of modified Dresden (Siena) protocol [18] and accelerated 9 mW CXL in preventing and halting progression of KC over a 10-year period [18, 19, 25]. Specifically, in pediatric age group, CXL has good reported efficacy in halting KC progression. A long term prospective evaluation by Henriques et al. reported effective halting of KC progression after CXL in majority of the eyes and CXL failure rate (defined as increase of >1 D in Kmax after 1 year of CXL) as 23.07% over a period of three years in pediatric patients [26]. Management protocols for pediatric KC also differ in different geographic regions. While some surgeons advocate CXL early in children, others wait for progression before offering CXL. In the current study, we followed the global consensus guidelines for management of pediatric KC cases and evaluated the outcomes with these management guidelines. Using these guidelines, we were able to achieve good outcomes in all subgroups of pediatric KC including stabilization of KC in 83.3% of the eyes with the use of CXL. CXL with accelerated protocol reduces surgical time in pediatric patients, as compared to the standard Dresden protocol. Nearly half (∼43.1%) of pediatric KC cases were found to be progressive in the current study. The authors, therefore, recommend an early diagnosis of KC and prompt CXL treatment in pediatric KC.

KC eyes with concomitant VKC often present with a progressive disease, due to ongoing inflammation [27]. This is supported by evidence of raised matrix metallopreoteinases (MMP-9) levels in tear films of eyes with KC and VKC [27]. We also found that the presence of VKC did not affect the success of CXL. Similar results were reported with use of standard (Dresden protocol) CXL in pediatric KC eyes with VKC by Stone et al. [28] in a two-year retrospective case-control study. The authors noted failure rate in 18.5% in VKC group and 16.7% in non-VKC group (*p*=0.83). In our study, 12% of the patients in VKC group and 11.8% of the patients in non-VKC group continued to progress at two years after CXL (*p*=0.39). In contrast, Shetty et al. [29] reported a higher failure rate (17.65%) of CXL in pediatric KC with VKC as compared to those without VKC. They concluded that management of VKC prior to CXL would reduce ocular inflammation and could also improve the success of CXL [29]. The difference may be explained by higher number of cases of VKC in our study and the fact that all our cases of VKC underwent CXL only after adequate control of inflammation.

There is a global consensus that CLs and scleral lenses are important for visual rehabilitation in the management of pediatric KC [7]. These do not halt the KC progression. For mild KC, soft or soft toric lenses are tried, but for severe cases, the rigid gas permeable (RGP) lenses are the most commonly used lenses. In severe KC, CL fitting becomes a challenge due to steep cones in these eyes. CL intolerant cases may have to be taken up for keratoplasty surgery. Rose K lenses have been reported to be successful in more than 90% of KC cases [30, 31]. However, very little is known about outcomes of CL in pediatric KC. In our study, although contact lenses were used in a small proportion of patients, there was a significant improvement in visual acuity in this subset of patients.

Corneal transplantation is indicated for cases of KC with corneal scarring, unsatisfactory vision, poor CL fit, or CL intolerance. Overall, a trend towards decreased rates of corneal transplantation for keratoconus has been reported. PK and DALK are surgical options for pediatric keratoconus. DALK has several advantages over PK, especially for pediatric patients, including elimination of endothelial graft rejection, preservation of globe integrity, and less stringent donor cornea criteria [22]. As case selection for DALK requires absence of full thickness scarring, there are limited cases indicated for DALK. In a large pediatric cohort study, Feizi et al. evaluated outcomes of DALK performed by big-bubble technique in pediatric KC and reported promising results. Both DALK and PK were performed in our study as per the guidelines from the global consensus. The short term outcomes were good after both types of surgeries.

Our study has some limitations like a relatively short follow-up period (two years) and a small sample size in each intervention group. Although Amsler–Krumeich classification was stated as outdated in the global consensus guidelines, yet it was used in our study to see clinical response in different severity grades. But when the VKC and non-VKC groups were stratified based on KC stage, the clinical effect was difficult to ascertain, as the number of cases in each stage was very small. The global consensus guidelines do not recommend any particular protocol of CXL: Dresden or accelerated. Larger case-control studies may be undertaken to evaluate the impact of VKC on the long term outcomes of ACXL. However, the result of this study showed good utility of recommendations of global consensus guidelines in the management of pediatric keratoconus.

## Figures and Tables

**Table 1:** Treatment outcomes of pediatric keratoconus with different management modalities using global consensus guidelines.

Parameter	Baseline	12 months	24 months	*p* value
*(A) Crosslinking group*				
Spherical equivalent (D)	−4.58 ± 3.14	−4.42 ± 3.05	−4.46 ± 3.12	0.76
Refractive astigmatism (D)	3.9 ± 2.62	3.85 ± 2.6	3.89 ± 2.58	0.85
K mean (D)	50.46 ± 4.31	49.45 ± 4.45	48.83 ± 4.62	<0.001^*∗*^
K max (D)	57.86 ± 7.43	56.6 ± 6.4	56.05 ± 6.29	<0.001
Thinnest pachymetry (*μ*m)	432.3 ± 45.64	419.45 ± 46.69	421.67 ± 48.64	0.178
Central pachymetry (*μ*m)	438.5 ± 37.4	434.64 ± 35.39	436.7 ± 36.23	0.011^*∗*^
Higher order total aberrations (*μ*m)	1.702 ± 1.56	1.26 ± 1.31	1.10 ± 1.19	<0.001^*∗*^
Coma (*μ*m)	1.007 ± 0.88	0.96 ± 0.88	0.95 ± 0.72	0.11
Trefoil (*μ*m)	0.82 ± 0.75	0.54 ± 0.64	0.45 ± 0.6	<0.001^*∗*^
Basal cell density (in cells/mm^2^)	3295.10 ± 363.22	3390.19 ± 343.80	3129.92 ± 289.9	0.456
Endothelial cell count (cells/mm^2^)	3036.3 ± 388.13	3028 ± 384.91	2977.47 ± 469.95	0.429
Anterior stromal keratocyte density (cells/mm^2^)	1963.72 ± 198.54	1939.76 ± 215.42	1943.97 ± 206.93	0.899
Middle stromal keratocyte density (cells/mm^2^)	1936.5 ± 204.88	1939.85 ± 198.7	1902.72 ± 367.46	0.631
Posterior stromal keratocyte density (cells/mm^2^)	1996.72 ± 202.95	1970.69 ± 179.12	1992.14 ± 186.93	0.791
CRF (mm Hg)	6.78 ± 1.5	6.6 ± 1.3	6.5 ± 1.38	0.194
CH (mm Hg)	7.73 ± 1.44	7.5 ± 1.3	7.45 ± 1.41	0.228

*(B) Spectacles group*				
BSCVA, in logMAR units	0.72 ± 0.54	0.62 ± 0.7	0.61 ± 0.7	0.187
MRSE, in D	−2.74 ± 3.29	−2.735 ± 3.22	−2.729 ± 3.25	0.181
Refractive astigmatism, in D	2.38 ± 1.73	2.32 ± 1.71	2.31 ± 1.66	0.07

*(C) Contact lenses group*				
BCLCVA, logMAR units	0.23 ± 0.22	0.17 ± 0.06	0.17 ± 0.6	<0.001^*∗*^
MRSE (dioptres)	−4.86 ± 3.89	−4.7 ± 4.09	−4.45 ± 4.88	0.11
Mean K	49.27 ± 4.19	48.46 ± 4.36	48.47 ± 4.44	0.07

*(D) DALK group*				
UCVA, logMAR units	1.53 ± 0.27	0.32 ± 0.04	0.31 ± 0.04	<0.001^*∗*^
BCVA, logMAR units	1.24 ± 0.46	0.16 ± 0.08	0.14 ± 0.09	<0.001^*∗*^
MRSE (D)	−6.1 ± 5.82	−1.28 ± 1.2	−1.08 ± 1.1	0.01^*∗*^
Mean astigmatism (D)	4.81 ± 2.38	1.34 ± 1.63	1.3 ± 1.4	0.001^*∗*^
Mean K (D)	64.31 ± 8.53	46.78 ± 5.9	46.2 ± 7.5	<0.001^*∗*^

*(E) PK group*				
UCVA, logMAR units	1.67 ± 0.21	0.38 ± 0.18	0.37 ± 0.19	*p* < 0.001^*∗*^
BCVA, logMAR units	1.54 ± 0.4	0.09 ± 0.08	0.08 ± 0.12	*p* < 0.001^*∗*^
MRSE, in dioptres	−8.45 ± 3.73	−3.14 ± 1.36	−2.92 ± 1.25	*p*=0.004^*∗*^
Mean astigmatism	5.75 ± 2.29	2.13 ± 1.58	2.04 ± 1.58	*p* < 0.001^*∗*^
Mean K	66.3 ± 6.98	47.44 ± 1.73	47.04 ± 0.956	*p* < 0.001^∗^

*(F) Intracameral C*_*3*_ *F*_*8*_ *gas injection*				
BCVA logMAR units	1.84 ± 0.27	1.34 ± 0.52	1.346 ± 0.52	<0.001^*∗*^
UCVA, logMAR units	1.84 ± 0.27	1.46 ± 0.46	1.41 ± 0.04	0.001^*∗*^
CCT, in *μ*m	1915.77 ± 373.6	518.5 ± 22.99	479.33 ± 26.39	<0.001^*∗*^

D: dioptre, K: keratometry, UCVA: uncorrected visual acuity, BSCVA: best spectacle-corrected visual acuity, MRSE: mean refractive spherical equivalent, BCLCVA: best contact lens corrected visual acuity, PK: penetrating keratoplasty, DALK: deep anterior lamellar keratoplasty, CCT: central corneal thickness, CRF: corneal resistance factor, and CH: corneal hysteresis. ^*∗*^Significant *p* value (<0.05). More than one type of intervention was used in some patients.

## Data Availability

Data are available upon request.
